# Determination of Ligand Binding Modes in Hydrated Viral Ion Channels to Foster Drug Design and Repositioning

**DOI:** 10.1021/acs.jcim.1c00488

**Published:** 2021-07-27

**Authors:** Balázs Zoltán Zsidó, Rita Börzsei, Viktor Szél, Csaba Hetényi

**Affiliations:** † Pharmacoinformatics Unit, Department of Pharmacology and Pharmacotherapy, Medical School, University of Pécs, Szigeti út 12, 7624 Pécs, Hungary; ‡ Department of Pharmacology, Faculty of Pharmacy, University of Pécs, Szigeti út 12, 7624 Pécs, Hungary

## Abstract

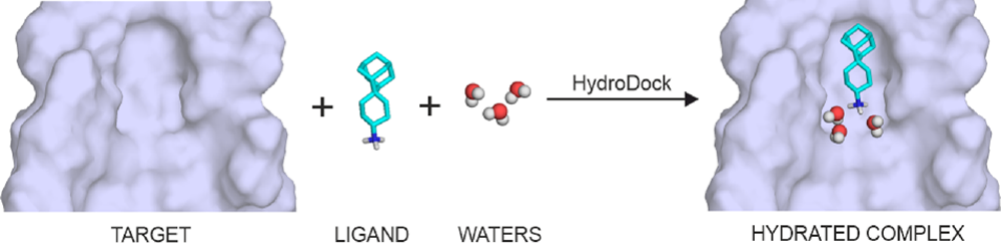

Target-based design and repositioning are mainstream strategies of drug discovery. Numerous drug design and repositioning projects have been launched to fight the ongoing COVID-19 pandemic. The resulting drug candidates have often failed due to the misprediction of their target-bound structures. The determination of water positions of such structures is particularly challenging due to the large number of possible drugs and the diversity of their hydration patterns. To answer this challenge and help correct predictions, we introduce a new protocol HydroDock, which can build hydrated drug–target complexes from scratch. HydroDock requires only the dry target and drug structures and produces their complexes with appropriately positioned water molecules. As a test application of the protocol, we built the structures of amantadine derivatives in complex with the influenza M2 transmembrane ion channel. The repositioning of amantadine derivatives from this influenza target to the SARS-CoV-2 envelope protein was also investigated. Excellent agreement was observed between experiments and the structures determined by HydroDock. The atomic resolution complex structures showed that water plays a similar role in the binding of amphipathic amantadine derivatives to transmembrane ion channels of both influenza A and SARS-CoV-2. While the hydrophobic regions of the channels capture the bulky hydrocarbon group of the ligand, the surrounding waters direct its orientation parallel with the axes of the channels via bridging interactions with the ionic ligand head. As HydroDock supplied otherwise undetermined structural details, it can be recommended to improve the reliability of future design and repositioning of antiviral drug candidates and many other ligands with an influence of water structure on their mechanism of action.

## Introduction

The COVID-19 pandemic has generated a tsunami in target-based drug design^1^ and repositioning.^2^ Target-based design is a widely used approach^3−7^ where the target structure serves as a reference point for fitting and selection of drug candidates. Repositioning is a cheap and fast strategy of drug discovery, as the pharmacological profile of known drugs is readily available with detailed information on their pharmacodynamics, pharmacokinetics, toxicity, interactions, and side effects. The clinical repositioning trials of a number of known drugs were launched in the past year^8−11^ to test their applicability against the severe acute respiratory syndrome coronavirus 2 (SARS-CoV-2). Although a few drugs were approved for clinical use, the repositioning trials have not led to real breakthroughs against SARS-CoV-2.

The failure of repositioning trials can be largely attributed to the structural differences between the old and new targets. For example, the structural dissimilarities between the active sites of proteases of HIV-1 and SARS-CoV-2 forecasted^12,13^ the failure of recent repositioning trials^8,14^ of HIV-1 protease inhibitors lopinavir and ritonavir to SARS-CoV-2. Such painful lessons highlight the necessity of a careful structure-based design and repositioning to reduce the number of failed clinical trials.

**Figure 1:**
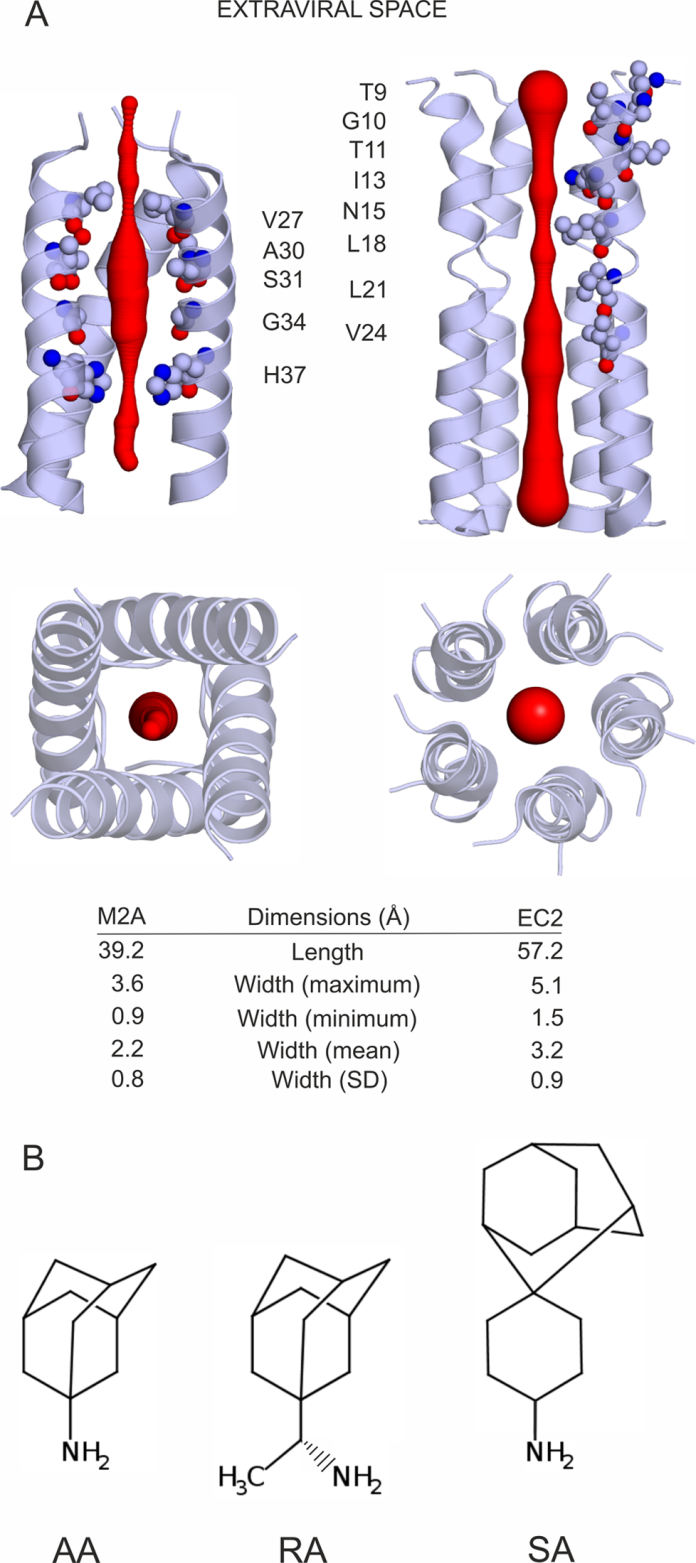
(A) M2A (left) and EC2 (right) ion channels shown as cartoon. The red cone represents the diameter of the ion channels. Interacting amino acids are labeled and shown as spheres in the side views at the top (a helix was deleted to show the interior of the channels). Top views from the extraviral space and lists of dimensions of the channels are shown at the bottom. (B) Lewis structures of the three AA derivatives investigated in the present study. Under physiological conditions, the amino group is protonated, resulting in a net charge of +1. *R*-rimantadine was used in the study, referred to as RA.

In the present study, we investigate the structural basis of repositioning of FDA-approved drugs amantadine (AA, Gocovri, Symmetrel) and its derivatives, rimantadine (RA, Flumadine) and spiroadamantyl amine (SA),^15−20^ (Figure 1b) to the ion channel formed by the transmembrane domain of the SARS-CoV-2 envelope protein (EC2, Figure 1a) as a possible “new” target. These AA derivatives were shown to inhibit the cation conductance of the M2 transmembrane ion channel of influenza A virus (M2A, Figure 1a),^21^ and the “old” target was also used as a reference in this study. AA was originally suggested^16^ against SARS-CoV and showed various beneficial effects in patients infected by the SARS-CoV-2^18−20^ as well. EC2 is homologous to the envelope protein of SARS-CoV^16^ and also functions as a cation-selective ion channel like M2A, playing a role in virus budding, release, and host inflammation response.^15^ The blocking of EC2 by AA derivatives or similar amphipathic molecules is a promising drug design strategy^22,23^ even on a longer term due to the low mutagenicity of EC2 found in mutated SARS-CoV-2 lineages collected from patients in India.^24^ Recently, the atomic resolution structure of EC2 (Figure 1a) was determined^15^ using solid-state NMR, providing a starting point for target-based design. The same study demonstrated the binding of fluorinated AA to EC2 as well. The large pore^15^ of EC2 is formed in a pentameric helical bundle stabilized by interhelical aromatic stacking interactions.

The pore size of EC2 is comparable to that formed by the tetrameric bundle in M2A^21^ (Figure 1a), which captures the AA derivatives. The similar pore geometry of M2A and EC2 is just one structural factor if considering the repositioning of ligands between the two ion channels.

Their amino acid composition and water structure^25^ are also key factors of ligand binding. The mediating role of water molecules was highlighted in the binding mechanism of AA derivatives to M2A.^21,25^ Considering the above similarity between M2A and EC2, one may expect that understanding the role of water molecules will be important in the case of EC2 as well. The available EC2 structure^15^ is an apo form without water and ligand molecules (Figure 1a), and therefore, it cannot supply any information on the possible mediating role of water molecules in ligand binding to EC2. Thus, an atomic resolution structure of the full complex with a bound ligand and water molecules (a hydrated holo structure) is necessary to foster correct repositioning and design to EC2.

As the full complex has not been solved at atomic resolution, we have to calculate the binding of the AA derivatives and the water structure from scratch, which is a challenging task for current methods.^25^ To answer this challenge, we introduce a new protocol that will supply the water structure of the EC2 channel and also adopt docking and molecular dynamics steps to produce the representative binding modes of AA derivatives. The protocol will be tested on the old M2A target with available experimental complex structures as references and will be transferred to the new EC2 target. In this way, we will explore the role of water in binding of the AA derivatives and produce their key binding modes on the new EC2 target, supplying the necessary atomic resolution structures for repositioning and design.

## Methods

### Input Structures

The atomic coordinates of M2A complexed with AA (6BKK), RA (6BKL), and SA (6BMZ)^21^ and the ligand-free structure of M2A (3LBW)^26^ were acquired from the Protein Databank (PDB). A, B, C, and D chains and their corresponding ligand (except for the apo structure) and water molecules were used for protocol development and validation purposes (Sections “The Effect of Interfacial Water Molecules on Ligand Docking to the Influenza A M2A Channel” and “Construction of the Ligand-Bound, Hydrated Influenza A M2A Channel Structures from Scratch”). The EC2 NMR structure (first model of the 20) from ref (15) (7K3G) was used in Section “Ligand Binding Modes and the Water Structure in the EC2 Channel of SARS-CoV-2” to create the hydration structure and ligand binding modes from scratch.

#### Ligand Preparation

Ligands were built in Maestro.^27^ The raw structures were energy-minimized using a semiempirical quantum chemistry program package, MOPAC^28^ with PM7 parametrization.^29^ The gradient norm was set to 0.001. The energy-minimized structures were submitted to force calculations; the force constant matrices were positive definite. Restrained electrostatic potential (RESP) charges were calculated with RED-vIII.52^30^ after geometry optimization by GAMESS.^31^ Acpype^32^ and antechamber^32,33^ were used to assign bound parameters and atom types for topology of ligands.

#### Target Preparation

The N-terminal ends of the ion channels were capped with acetyl groups and the C-terminal ends with imino-methyl groups using Maestro^27^ and were subjected to energy minimization in the merging step (Step 3). Hydrogen atoms and Gasteiger–Marsili partial charges^34^ were added to the targets with AutoDock Tools.^35^ After ligand and target preparation, the dry target and respective ligands were used as starting points of HydroDock (next section).

### HydroDock

**Figure 2:**
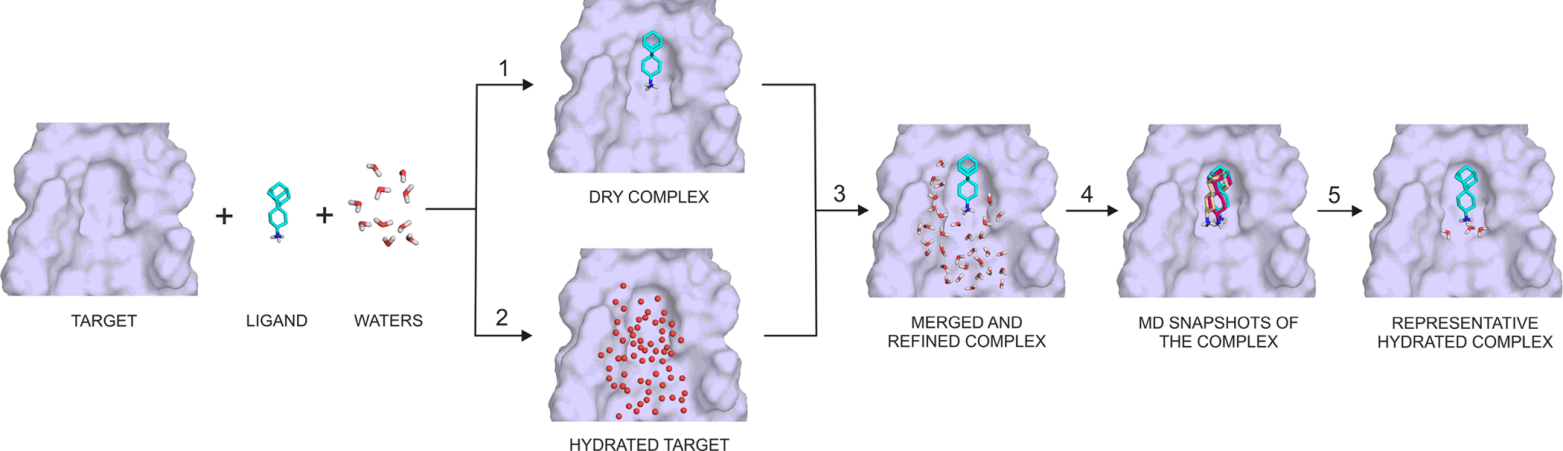
Assembly of the hydrated complex of the M2A channel (target, surface) and SA (ligand, sticks) from scratch using the HydroDock protocol. The numbering of steps of HydroDock follows the explanation in the main text. After the first step, nonminimized water positions from MobyWat^40,41^ are shown as red spheres; otherwise, sticks representation is used for hydrogenated and minimized waters. During the third step, some of the water positions are replaced by the ligand. For clarity, only a few MD snapshots of ligand binding modes are shown after the fourth step. Coordinate files of all snapshots are accessible in the Supporting Information.

HydroDock is a new protocol shortly featured in Section 2 of Results and Discussion. The steps of HydroDock are numbered in Figure 2 and referred to in the following detailed descriptions using the same numbering as in Results and Discussion.

#### Step 1. Dry Docking

Blind docking was performed as described before^36^ for both targets M2A and EC2 (Box B, Table S5). During blind docking, the docking box covered the whole surface of the target. Focused docking was also used for EC2 when the box only covered the upper half of the protein (Box A, Table S5). The unliganded M2A and EC2 structures were used as targets of the blind and focused docking runs. No explicit water molecules were adopted from the PDB structures. The target was treated as a rigid body except that the flexibility of the N15 amino acid side chains was allowed on all helices of EC2 to allow the entrance of the ligand toward the intraviral regions (Table S5). AutoGrid 4.2^35^ was used for grid map calculations. Grid boxes were generated around the entire M2A target. The grid boxes were centered on the target, and 70 (M2A) and 90 (EC2) grid points along all axes were set with 0.503 Å grid spacing (0.375 Å in Box A). The resulting docking box covered the entire M2A and EC2 in the case of blind docking and allowed the entrance of the ligands from both extra- and intraviral regions. To avoid artefacts and allow ligand entrance only from the extraviral space (Figure 1a), the docking box was reduced to only cover the upper half of EC2 (Box A, Table S5).

Molecular docking calculations were performed by AutoDock 4.2.^35^ Hydrogen atoms and Gasteiger–Marsili^34^ partial charges were added to the ligands with an OpenBabel^37^ program package. All chemically relevant torsions of the ligands were enabled. One hundred blind docking runs were performed. The Lamarckian genetic algorithm and the pseudo-Solis and Wets local search with a maximum number of 300 iterations and 25 million energy evaluations and 150 population size were applied as in refs (38) and (39). The generated 100 ligand binding modes were clustered and ranked (see Section “Evaluation Criteria” for details) based on their calculated free energy of binding values and structural similarity. Representative ligand structures of each rank in complex with their dry target structures were used as dry complexes. Due to the symmetry of both M2A and EC2, from among identical, symmetry-related rank representatives, the one with the lowest calculated binding free energy was selected and forwarded to the next steps of HydroDock.

In the case of M2A, a total of six representatives were found, one–one for all three AA derivatives on both holo and apo target forms (Table 1). In the case of EC2, five (AA1, ..., AA5, Table S5), two, and one representatives of AA, RA, and SA were found (eight in total) and forwarded to Step 3.

**Table 1:** Comparison of Computationally Docked and Experimental Binding Positions of Ligands AA, RA, and SA to a Dry M2A Target

ligand	M2A conformation	RMSD (Å)	rank^a^
AA	holo	3.3	1/1
AA	apo	3.7	1/1
RA	holo	3.8	1/2
RA	apo	3.6	1/1
SA	holo	4.8	1/3
SA	apo	2.9	1/1
mean		3.7	
SD		0.7	

a Serial number of rank/count of all ranks.

#### Step 2. Building the Water Structure of the Inner Surface of the Target Channels

The water structure of the inner surface of the target channels was built using MobyWat,^40,41^ which requires an MD trajectory of a target in explicit water as an input. The MD-based evaluation of MobyWat allows consideration of all solute–water and water–water interactions and results in high success rates if compared with experimental structures.^40,41^

##### Generation of MD Trajectories

The dry M2A (6BKK) and EC2 (7K3G) targets were energy-minimized by steepest descent and conjugate gradient algorithms as in Step 3 of HydroDock to prepare them for the 1 ns-long MD simulations. The simulation box was filled with explicit TIP3P^42^ water molecules, and counterions (sodium or chloride) were added to neutralize the system. Exit tolerance levels were set to 10^3^ and 10 kJ·mol^–1^·nm^–1^, while maximum step sizes were set to 0.5 and 0.05 nm for the steepest descent and conjugate gradient steps, respectively. Position restraints were applied on solute heavy atoms at a force constant of 10^3^ kJ·mol^–1^·nm^–2^. Calculations were performed with programs of the GROMACS^43^ software package using the AMBER99SB-ILDN^44^ force field. After energy minimization, 1 ns-long NPT MD simulation was carried out with a time step of 2 fs. For temperature coupling, the velocity rescale^45^ algorithm was used. The solute and solvent were coupled separately with a reference temperature of 300 K and a coupling time constant of 0.1 ps. Pressure was coupled by the Parrinello–Rahman algorithm^46,47^ and a coupling time constant of 0.5 ps, compressibility of 4.5 × 10^–5^ bar^–1^, and reference pressure of 1 bar. Particle mesh-Ewald summation^48^ was used for long-range electrostatics. Van der Waals and Coulomb interactions had a cutoff at 11 Å. Coordinates were saved at regular time intervals of 1 ps, yielding 1 × 10^3^ frames. Position restraints were applied on solute heavy atoms at a force constant of 10^3^ kJ·mol^–1^·nm^–2^. Periodic boundary conditions were treated before analysis to make the solute whole and recover hydrated solute structures centered in the box. Each frame was fit to the original protein crystal structure using Cα atoms. The final trajectory including all atomic coordinates of all frames was converted to portable XDR binary files equipped with name extension xtc.

##### MobyWat Calculations

From the MD trajectory, surface water positions were calculated with Mobywat’s^40^ all-inclusive identity-based (IDa) prediction algorithm. The maximum distance from the target (*d*_max_), prediction, and clustering tolerances were set to 5, 2.5, and 1 Å, respectively. The MobyWat algorithm was described earlier.^40,41^ Briefly, candidate water molecules for all frames are selected based on a desired distance limit (*d*_max_) from the target, and then an occupancy list is constructed containing every different water IDs on every line and the respective number of occurrences as candidates among all frames. Clustering is applied to all rows (all different water IDs) of the occupancy list using the ctol parameter to define the distance between elements of the same cluster. The largest cluster is selected from all to give the first predicted water molecule by averaging the spatial coordinates of included molecules. In the further steps, clusters are selected in a descending order size-wise and checked if their distance is larger than the prediction tolerance from previously predicted water positions. After the above clustering, a list of water positions (prediction list) was produced as the O atom coordinates covering the surface of the EC2 (7K3G) and M2A (6BKK) channels. The hydrogens were added to the predicted water O atoms in a later step (Step 3 of HydroDock).

In the case of M2A, the predicted water oxygen positions were compared to the reference water molecules in the PDB structure 6BKK using the validation mode of MobyWat. The above settings were used with a match tolerance of 1.5 Å.

#### Step 3. Merging and Refinement

##### Merging

The outcomes of Steps 1 and 2 were combined to build the raw complex structures, that is, the hydrated, ligand-bound targets. For this, the complexes were placed in a common coordinate system by alignment of the target structure of the dry complex from Step 1 and the hydrated target structure from Step 2 using PyMol.^49^ After alignment, a raw complex still contains all surface water molecules predicted by MobyWat. However, after the placement of the dry docked ligand structure into the fully hydrated target, some water molecules overlap with the ligand. The overlapping water molecules were removed by the editing mode of MobyWat,^40^ and only interfacial water molecules were retained. The merged structures (see Step 1. Dry Docking) of the eight EC2 complexes (Table S5) and six M2A complexes (Table 1) were then subjected to robust refinement.

##### Soft Refinement (Not Part of HydroDock and Used during Protocol Development) (Figure S1)

The interfacial crystallographic water oxygen atoms within a *d*_max_ of 5.0 Å distance limit from both the ligand and the target were kept, as they bridge between the ligand and the amino acid residues of the protein; other waters were removed. The structure of the M2A channel with the water O atoms was placed in a dodecahedral box using a distance criterion of 1 nm between the solute and the box. Void spaces of the box were filled by explicit TIP3P water molecules by GROMACS.^43^ Hydrogen atoms were added to water oxygen and solute atoms by the GROMACS program pdb2gmx. The system was neutralized by counterions. A steepest descent (steepest descent1) optimization was carried out,^40^ with convergence threshold set to 10^3^ kJ·mol^–1^·nm^–1^ followed by a conjugate gradient (conjugate gradient1) calculation, where the convergence threshold was set 10 kJ·mol^–1^·nm^–1^. Position restraints at a force constant of 10^3^ kJ·mol^–1^·nm^–2^ were applied on all heavy atoms in both steps. An AMBER99SB-ILDN^44^ force field was used for the calculations. The steepest descent and conjugate gradient minimization steps were carried out once again (steepest descent2, conjugate gradient2), with the same settings^40^ as in steepest descent1 and conjugate gradient1, with the exception that only backbone Cα atoms were position restrained.

##### Robust Refinement Was Adopted as an Appropriate Protocol of HydroDock Based on the Good Docking Results (Table 3)

Robust refinement has only one difference when compared to soft refinement; the steepest descent1+conjugate gradient1 step is not immediately followed by the steepest descent2+conjugate gradient2 steps, but first, a 100 ps-long MD simulation (md) is carried out (steepest descent1+conjugate gradient1+md+steepest descent2+conjugate gradient2). In the MD simulation, only backbone Cα atoms were position-restrained. Notably, in a general application of HydroDock for systems with large flexibility on the target backbones, the use of a membrane model would be advisable instead of position restraining of the backbone. For temperature coupling, the velocity rescale algorithm was used. The solute and solvent were coupled separately with a reference temperature of 300 K and a coupling time constant of 0.1 ps. Pressure was coupled with the Parrinello–Rahman algorithm with a coupling time constant of 0.5 ps, compressibility of 4.5 × 10^–5^ bar^–1^, and reference pressure of 1 bar. Particle mesh-Ewald summation was used for long-range electrostatics. Van der Waals and Coulomb interactions had a cutoff at 11 Å. Robust refinement resulted in the correct position of the experimental water molecules of M2A, with the right orientation of H atoms that led to the formulation of two water networks. Based on the success, robust refinement was adopted in Step 3 of HydroDock after merging.

##### Wet Docking (Not Part of HydroDock and Used during Protocol Development) to Choose the Sufficient Refinement Protocol and Validate It

In wet docking, every detail was set as in dry docking (Step 1 of HydroDock) except that refined water molecules were included. When compared to the experimental ligand positions, the Gasteiger–Marsili partial charges on the atoms of the water molecules yielded incorrect results (Figure S2). Thus, partial charges of the TIP3P explicit water model were used on all water molecules instead.

#### Step 4. Generating MD Snapshots of the Target–Ligand Complex

The MD simulations of the merged and refined complexes were carried out with the same settings described in the minimization procedure (robust refinement). The simulations were performed as listed in Table S4 and for 100 ns in the cases of M2A and EC2, respectively. Only the Cα atoms of the proteins were restrained. The movements of the amino acid side chains, the ligand, and the solvent were allowed. The refined hydration structure was kept in the MD simulations; the rest of the simulation box was filled with water molecules by GROMACS. Complex snapshots were aligned by a GROMACS tool trjconv using their target Cα atoms, and the bound ligand snapshots were separately generated as individual files from the MD trajectory file by 0.1 ns steps (conformation pool).

#### Step 5. The Selection of the Representative Ligand Binding Modes from the MD Trajectory File

An average ligand conformation was calculated from the conformation pool using a shell script provided in the Supporting Information file. RMSD values between the individual ligand pool structures and the average ligand pool structure were calculated according to eq 1, where the average pool conformation was used instead as a reference **C** in this case. A pool structure with the lowest RMSD value was selected as the representative ligand binding mode from the MD trajectory. A representative binding mode of the ligand is the suggested final binding mode to the target (M2A, EC2). Distinct binding modes produced by dry docking (Step 1) usually result in more than one representative structure after HydroDock.

### Evaluation Criteria

Standard criteria^50−54^ were applied to evaluate the results of dry and wet docking and HydroDock. In all cases, the structural match of the calculated (docked or HydroDock representative, **D** in eq 1) binding mode to the crystallographic reference (**C**) was expressed as a root-mean-square deviation (RMSD) value according to eq 1(51)
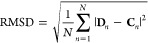
1In eq 1, *N* is the number of ligand heavy atoms, **C** is the space vector of the nth heavy atom of the crystallographic reference ligand molecule, and **D** is the space vector of the nth heavy atom of the calculated ligand conformation. Overlapping ligand conformations resulted by 120° turns around the trigonal vertical axis were considered identical during RMSD calculations.

The ranking order was also shown in the cases of dry and wet docking trials. The docked ligand conformations were structurally clustered and ranked according to their AutoDock 4.2 binding free energy values, and the serial numbers of ranks are listed in Results and Discussion. During this procedure, the ligand structure with the lowest calculated free energy of binding was selected, and the neighboring docked ligand structures within 2 Å^38^ were collected in the rank; then, a new rank is opened starting with an unused structure of the lowest calculated free energy of binding from the remaining structures, etc. until all 100 ligand structures were collected into ranks.^40^ Ranks with a low serial number indicate an energetically favorable binding conformation. Note that in the case of HydroDock, representative binding modes were selected (Step 5) without the need of further ranking.

### Calculation of Interaction Energy Values of SA-EC2 Complexes

The Lennard-Jones interaction energy (*E*_LJ_) was calculated between the target and ligand molecules according to eq 2
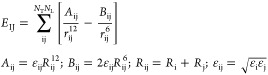
2In eq 2, ε_i_ and ε_j_ are the potential well depths in the equilibrium distance of atom pairs of identical types; ε_ij_ is the potential well depth in equilibrium between the ith (ligand) and jth (target) atoms; *R*_ij_ is the internuclear distance at equilibrium between ith (ligand) and jth (target) atoms; *R*_i_ and *R*_j_ are half equilibrium distances between ii and jj atom pairs of identical types, respectively; *r*_ij_ is the actual distance between the ith (ligand) and jth (target) atoms; *N*_T_ is the number of target atoms; and *N*_L_ is the number of ligand atoms. The Amber 2012 force field parameters were used.^56^ The calculations were performed for dry and hydrated targets as well. In the case of the hydrated target, explicit water molecules were considered as part of the target.

## Results and Discussion

### (1) The Effect of Interfacial Water Molecules on Ligand Docking to the Influenza A M2A Channel

**Figure 3:**
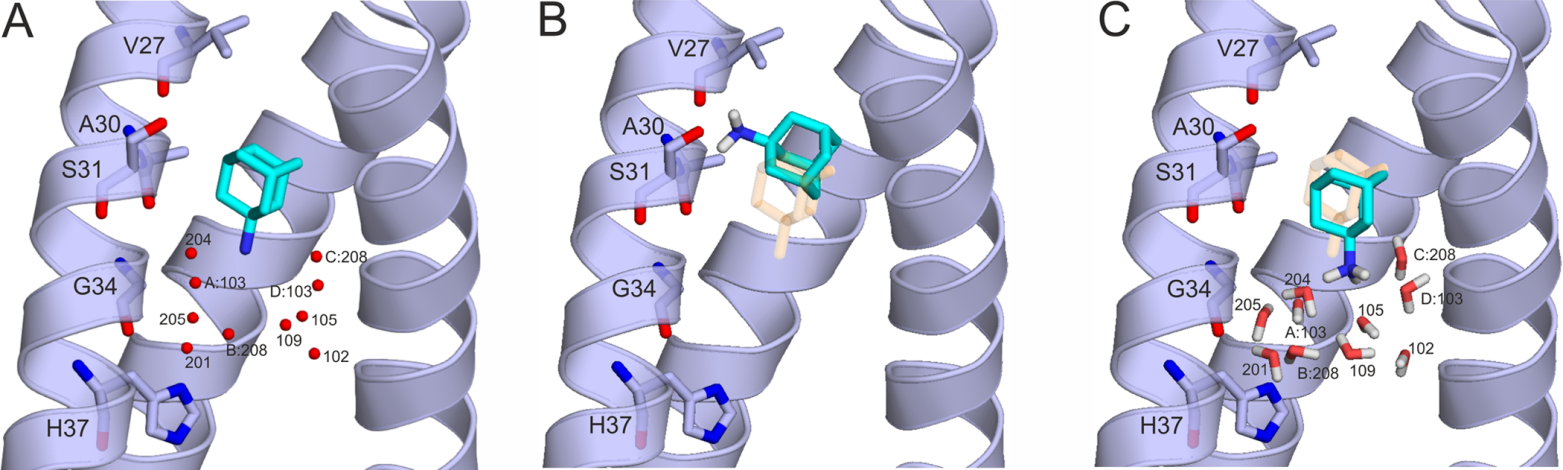
Complex of AA (sticks with teal carbon) bound to the M2A channel (cartoon and sticks with gray carbon, a frontal helix turned off for clarity). (A) Experimental binding mode in the PDB structure 6bkk with three H-bonds formed between the ligand protonated amino N and water O atoms. Water molecules are represented as red spheres and labeled by their chain IDs and/or residue numbers. (B) Result of “dry” docking of AA shows a shift of the positive protonated amino group of AA. Instead of the missing water molecules, interactions with the partially negative backbone carbonyl groups of V27 and A30 were formed. (C) Result of “wet” docking of AA is in a good agreement with the experimental binding mode of AA shown in panel A. The minimized water molecules are shown as thick lines and labeled according to the residue numbering in the PDB structure 6bkk. The crystallographic ligand binding position in (A) is also shown in (B) and (C) with transparent orange sticks for comparability.

Water molecules play a key role^21^ in binding AA and its derivatives to the influenza A M2A channel. For example, water (w) molecules A:w103, B:w204 (at A30) and B:w201, and C:w205 (at G34) form bridges between the positive protonated amino group of AA and the carbonyl oxygens of the amino acids (Figure 3a, the numbering of the PDB structure 6bkk is used). Together with other water molecules at H37, a static H-bonding network of 10 water molecules is formed, filling the channel cavity below AA (Figure 3a). Incorporation of such water molecules in docking calculations can be essential^57−60^ to obtain precise results.

To check this assumption, a systematic investigation of computational docking of all three ligands (AA, RA, and SA) was performed to the M2A channel using different approaches of handling interfacial water molecules. Targeting the dry M2A channel without any surface water molecules (Table 1) is the simplest approach and provides a basis for comparisons throughout this study. An average of 3.7 ± 0.7 Å root-mean-square deviation (RMSD) was calculated between the docked and crystallographic ligand conformations with the latter ones used as references. This value is above the RMSD of 1.5–2.0 Å considered acceptable in the literature^50−54^ and indicates that the dry M2A channel may not be an appropriate target for docking. The dry M2A channels in holo (ligand-bound) conformations did not yield significantly better results than the apo ones as docking targets. This follows from the high identity between the holo and apo target structures with an average superposition RMSD of 0.3 Å ± 0.1 (Table S2). Thus, there is no considerable induced fit during ligand binding to the M2A channel, and the rigidity (Methods) of the target structure did not affect the result in these cases. In the docked structure (AA-Holo in Table 1), the adamantyl group of AA was close to the crystallographic position (Figure 3b). However, the lack of the abovementioned (Figure 3a)^21^ bridging water molecules resulted in a miscoordination of the protonated amino group to the carbonyl oxygen of V27 and the hydroxyl group of S31 (Figure 3b) and the large RMSD values of Table 1.

In the wet docking calculations, a set of functional water positions (Table 2 and Figure 3c) of the crystal structures was used together with the M2A channel as a target. As the coordinates of water hydrogen atoms were not available, a theoretical refinement was necessary to add and optimize their positions. During the refinements, the ligand was kept in the holo structure to help in the correct arrangements of water hydrogen atoms in contact with the protonated amino group.

**Table 2:** Deviations of Refined Crystallographic and MobyWat-Predicted Water Positions Used in Wet Docking Calculations Measured from the Original Crystallographic Positions (PDB ID 6bkk) with Their Close Contacts Also Listed

water #^a^	close contact^b^	soft refinement (Å)	robust refinement (Å)	predicted (Å)^c^
A:w102		0.3	0.2	0.2
A:w103		0.6	1.6	0.9
D:w103		0.5	0.5	0.9
D:w105	D:w109	0.7	2.1	0.6
D:w109	C:w208, D:w105	0.9	1.0	2.1
B:w201	B:G34	1.0	1.0	0.6
B:w204		0.8	1.6	0.7
C:w205		0.4	0.9	0.5
B:w208		0.8	0.8	1.0
C:w208	D:w109	0.9	0.2	0.3

a The numbering of PDB structure 6bkk is used (see Table S1 for details of selection of reference structures).

b Close contacts of the crystallographic structure were listed if the distance between the oxygen atom of the actual water molecule and a heavy atom of a neighboring residue or the oxygen of the neighboring water molecule was below 2.75 Å.

c Crystallographic water positions of PDB structure 6bkk were used as reference; see also Table S1 for details on selection of reference crystallographic structures.

Two refinement protocols (a soft and a robust one) were investigated. During the soft protocol (soft refinement), simple energy minimization steps were applied (Methods) for the water hydrogen atoms while the positions of all heavy atoms (including water oxygen) were restrained in their crystallographic positions. The docking of AA to the ligand-free, S-refined target still did not result in an acceptable RMSD (2.7 Å), which can be attributed to the incorrectly positioned water hydrogen atoms (Figure S1). A closer inspection of the S-refined target structure showed that the incorrect positioning of water hydrogen atoms was a consequence of several close contacts (Table 2) in the original crystallographic water structure.^21^ The close contacts were maintained by the position restraints during soft refinement, resulting in relatively small shifts from their crystallographic positions (Table 2), hindered reconstruction of the interfacial H-bonding network, and atomic positions preformed to interact with AA (Figure S1). As docking of AA to the wet M2A target with soft refinement did not improve the dry results (Table 1), a robust protocol was also tested (robust refinement) including a molecular dynamics step with no restraints on the atoms. Robust refinement appropriately shifted half of the water molecules of Table 2 (A:w103, D:w105, D:w109, B:w201, and B:w204) to 1 Å or a larger distance (Table S3) from their crystallographic positions. In this way, their erroneous close contacts were eliminated, and their hydrogen atoms were arranged into correct orientations, resulting in a perfect H-bonding network. Some experimenting with the partial charge system on water molecules showed that TIP3P^42^ outperformed Gasteiger–Marsilli^34^ partial charges (Figure S2). Robust refinement and TIP3P charges on water molecules yielded excellent docking results with an average RMSD of 1.2 ± 0.3 Å (Table 3) for all ligands. The low serial numbers/counts of the corresponding ranks indicate that the structural precision reflected by the low RMSD values was accompanied by the best calculated binding free energies (or a single, homogeneous rank was produced). In the case of AA, docking to the wet, apo M2A channel structure was also performed after robust refinement. Similar to the holo results, an excellent RMSD of 1.0 Å was obtained (Figure 3c).

**Table 3:** Comparison of Computationally Docked and Experimental Binding Positions of Ligands AA, RA, and SA to the M2A Target Covered by Crystallographic Water Positions Subjected to a Robust Refinement Protocol and Equipped with Partial Charges of the TIP3P Explicit Water Model

ligand	M2A conformation	RMSD (Å)	rank^a^
AA	holo	1.2	1/1
AA	apo	1.0	1/1
RA	holo	1.0	1/2
SA	holo	1.7	1/1
mean (holo)		1.2	
SD (holo)		0.3	

a Serial number of the rank/count of all ranks.

The results of Table 3 showed that appropriately placed and oriented water molecules are keys to precise docking results if compared with the insufficient outcomes of dry docking (Table 1). It was also found (Table 2) that the availability of crystallographic water positions alone cannot guarantee the success for two reasons. (1) Often, only oxygen positions are supplied, and water orientations are obviously not assigned due to the lack of hydrogen atoms. (2) There are also other limitations^41,61−69^ of assignation of the crystallographic density map, resulting in missing or too many water molecules (overfitting). Such problems often result in crystallization artefacts^67^ and close contacts similar to those listed in Table 2. Thus, a robust theoretical refinement of experimental water structure is necessary in general and for correct calculation of complexes of all three ligands with the M2A channel in the present case.

### (2) Construction of the Ligand-Bound, Hydrated Influenza A M2A Channel Structures from Scratch

In agreement with other studies,^25^ the results of the previous section showed that docking calculations are very sensitive to even small errors in the water structure. In the previous examples (Table 2), a robust refinement of the measured water positions was necessary to achieve good docking results. In a real drug screening project,^36,55^ experiments cannot supply interfacial water positions and holo structures for all possible ligand molecules designed for the target binding pocket, and only an apo target structure is available for the docking calculations. Thus, only atomic coordinates of the individual components (ligand, target, and water) can be used for the construction work. It is a real challenge to bring all these partners together into a hydrated complex structure due to the difficulties of correct positioning of interfacial water molecules.^25^

To address this challenge, we introduce HydroDock, a hybrid protocol that supplies the hydrated complex structure from scratch. HydroDock is composed of five steps (Figure 2 and Methods) and was tested on the M2A target and its ligands (Figure 1). Step 1 involved a fast docking calculation with results described in Table 1. In Step 2, the water structure of the surface of the target was built by MobyWat^40,41^ with high precision. MobyWat is a molecular dynamics (MD)-based method that can predict solute–water and water–water interactions as well. In the present case, the inner surface of the M2A target was completely hydrated and the calculated water positions were compared to the crystallographic reference ones as listed in Table 2. Nine out of ten water molecules were successfully predicted at a match threshold of 1.0 Å (see also Figure S3). The predicted hydration structure was a priori close contact-free and equipped with hydrogen atoms, which is necessary for correct docking calculations (Section “The Effect of Interfacial Water Molecules on Ligand Docking to the Influenza A M2A Channel” and Table 3). In Step 3, the results of the first two steps were merged into one structure and surface water molecules overlapping with the docked ligand were eliminated using the Editing mode^40^ of MobyWat. In Step 4, the hydrated M2A–ligand complexes were subjected to molecular dynamics (MD) in a simulation box filled with explicit water molecules to generate a pool of several hundreds (*N*_pool_ in Table S4) of member conformations. Step 5 of HydroDock produces a representative complex conformation statistically selected from the pool (see Methods for the details of all steps).

**Figure 4:**
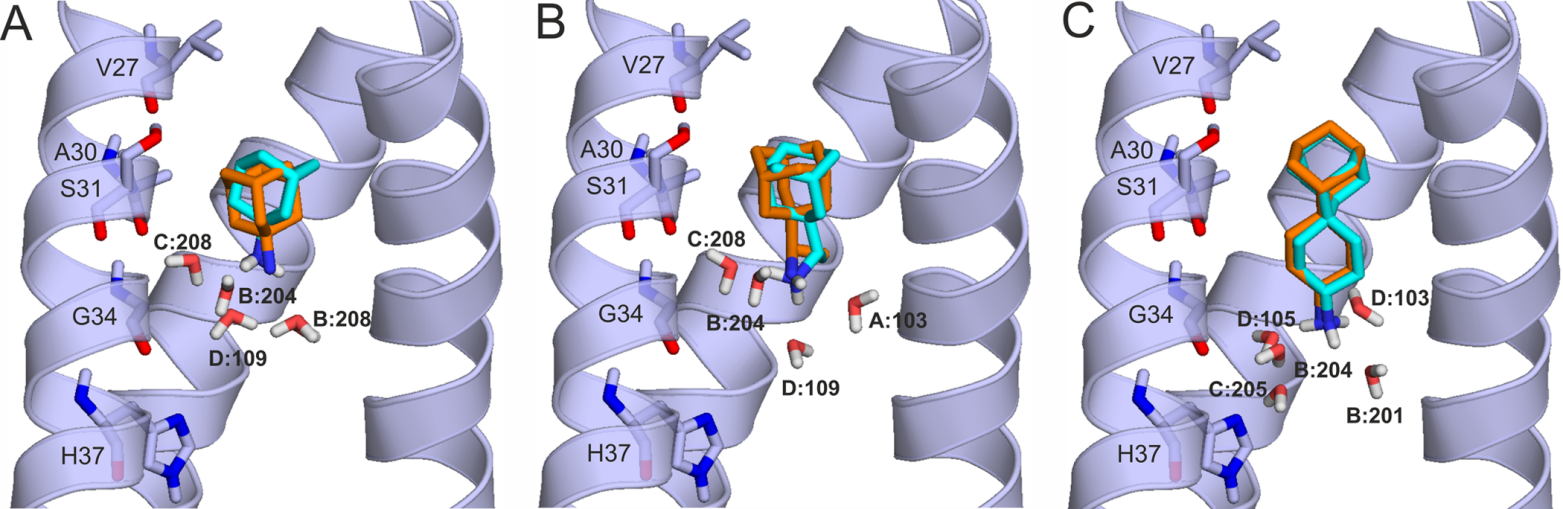
Representative binding modes of ligands (teal sticks for carbon atoms) (A) AA, (B) RA, and (C) SA in the complex with M2A (cartoon) produced by the HydroDock protocol. For comparison, crystallographic ligand binding modes (orange sticks for carbon atoms) are shown as references. Interacting M2A amino acids and water molecules are shown as sticks and labeled accordingly to the residue numbering of 6bkk.

The matches of the representative ligand conformations to the crystallographic ones are listed in Table 4 and shown in Figure 4. For these small ligands (Figure 1b), the conformation pools were generated in relatively short MD simulations of 40–100 ns (Methods and Table S4) appropriate for the selection of the representatives. The search space was also restricted by the helical boundaries of the narrow M2A channel (Figure 1a), and therefore, the selection of representatives was not particularly challenging from the ligand conformation pools containing fairly uniform binding modes (Table 4 and Table S4). Notably, in our previous study,^55^ we found that the generation of conformation pools in the cases of large, flexible ligands may require longer MD simulation times, especially if they bind to the target surface.

**Table 4:** Comparison of Computational and Experimental Binding Modes of Ligands AA, RA, and SA to the M2A Target^a^

ligand	M2A conformation	RMSD of representative (Å)	mean RMSD (Å)	SD RMSD (Å)
AA	holo	0.7	1.8	0.7
AA	apo	1.1	1.9	0.5
RA	holo	4.0	2.0	0.7
RA	apo	1.5	1.8	0.6
SA	holo	2.6	1.7	0.9
SA	apo	0.3	1.1	0.7

a The computational binding modes were produced by the HydroDock protocol introduced in the present study.

The final results (Table 4 and Figure 4) show excellent agreement with the experimental ligand conformations^21^ in all three cases. A closer inspection of the changes during the MD simulations (Step 4 of HydroDock) shows that ligand binding modes underwent considerable rearrangements due to their interactions with water molecules generated in Step 2. Due to the lack of the anchoring water molecules, dry docking (Step 1 of Hydrodock) produced misdocked binding modes exemplified by Figure 3a. During Step 4, all three ligands entered hydration networks of surrounding water molecules via their protonated amino groups that formed hydrogen bonds with water oxygen atoms (Figure 4). They also adopted their appropriate binding positions (Figure S4) with a rapid rotation and a slight downward movement toward the middle of the channel. Interestingly, besides the crystallographic binding mode, RA also adopted an alternative, parallel orientation corresponding to the higher RMSD of RA, holo in Table 4.

### (3) Ligand Binding Modes and the Water Structure in the EC2 Channel of SARS-CoV-2

A recent study^15^ explored the interactions of fluoro-AA with EC2 on the basis of chemical shift perturbations from nuclear magnetic resonance (NMR) spectroscopic measurements.

They also used docking calculations to map the anchoring residues during ligand entry from the extraviral space down to N15 of EC2 (Figure 1a).

**Figure 5:**
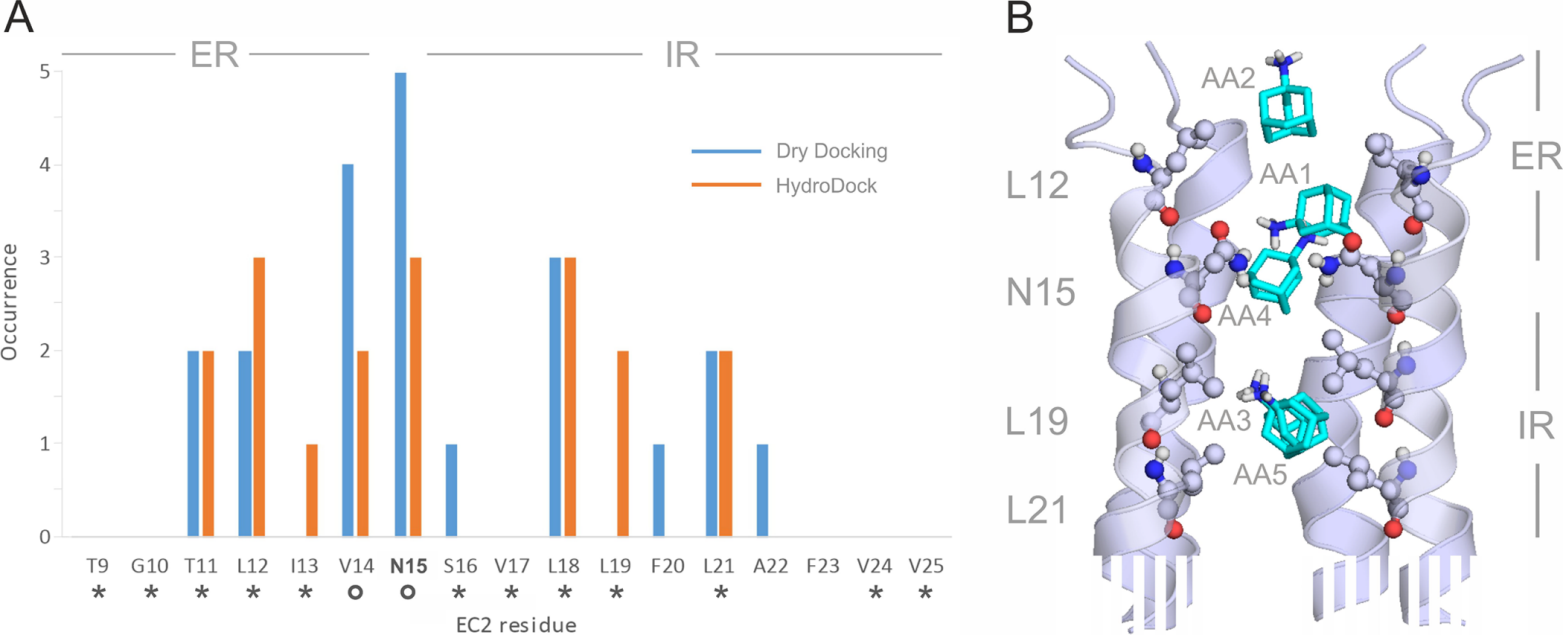
(A) Occurrence of EC2 amino acids interacting with AA in the five binding modes (bars) produced by dry docking (blue bars) and after refinement by HydroDock (orange bars) in the present study. Asterisks and circles indicate interacting amino acids identified by experiments and docking calculations, respectively, in a previous^15^ paper. Entrance and intrachannel binding regions are marked as ER and IR, respectively, at the top of the diagrams. (B) Five representative structures of binding modes AA1, ..., AA5 (teal sticks, Table S6) on EC2 (cartoon, truncated at the bottom). The interacting EC2 amino acids are shown as balls and sticks and labeled by their identifiers according to PDB structure 7k3g. ER and IR binding regions are also shown on the right side of the figure. Raw data are provided in Tables S5 and S6 in the Supporting Information.

The study identified a group of apolar entry residues T11...I13 by NMR (asterisks in Figure 5a) and others like N15 by docking calculations (empty circles), respectively. The fluoro-AA in ref (15) is only a slight modification of AA, both having a largely hydrophobic head group and a positively charged tail moiety (Figure 1b), and therefore, similar binding modes can be expected for both ligands on EC2.

Inspired by the above NMR-based study^15^ on EC2 and the good performance of HydroDock on the M2A channel (previous section), our protocol was applied to map the binding modes of the AA derivatives on the EC2 channel of SARS-CoV-2 (Figure 1). The binding modes of all three ligands (AA, RA, and SA) were mapped by HydroDock using the apo form EC2 as a target from ref (15). The interacting residues of EC2 were collected after dry docking (Figure 5a,b and Table S5) and for the final five representative binding modes produced by HydroDock (Figure 5a,b and Table S6) as well.

A good match was observed (Figure 5a) between the occurrence of EC2 residues involved in the binding modes of fluoro-AA identified in the NMR-based study^15^ and AA found by HydroDock in the present study. The results show two main binding regions (Figure 5a,b) of EC2, that is, an entrance region (ER) toward the extraviral space and an intrachannel region (IR) roughly divided by the gating residue N15. Our dry docking calculations showed that the IR region was accessible only in the case if the side chain of the gating N15 was free to move during the docking (Table S5 and Methods), indicating that N15 has a key role in ligand binding mechanisms. The NMR-based study^15^ also emphasized the role of this gating residue and concluded that small molecular drug candidates should show high binding affinity to N15 during their entry into EC2.

Water molecules significantly influence the binding modes of ligands to their targets^25^ (see also previous sections). As in the above M2A examples, HydroDock refinement of EC2 systems also involved structural hydration, energy minimization, and subsequent 100 ns-long MD simulations for all binding modes found in dry docking (Step 1 in Figure 2). The comparison of the binding pattern after dry docking (blue bars in Figure 5a) with that after HydroDock refinements (orange bars in Figure 5a) may shed light on the influence of water structure on ligand binding to EC2. The hydrophobic belts of binding modes AA1, AA2, and AA4 (ER) and AA3 and AA5 (IR) maintained after HydroDock refinements (Figure 5a). The ER and IR binding regions consist of hydrophobic cores centered on residues L12 (ER) and L19 and L21 (IR), respectively. While the hydrophobic interactions appear in both dry docking and HydroDock results (Figure 5a), there are certain amino acids like L19 found by only one of the methods. In these cases, a rearrangement of the H-bonding system around the protonated amino group of AA was observed further as discussed in the next section and in Figure S5 in details.

**Figure 6:**
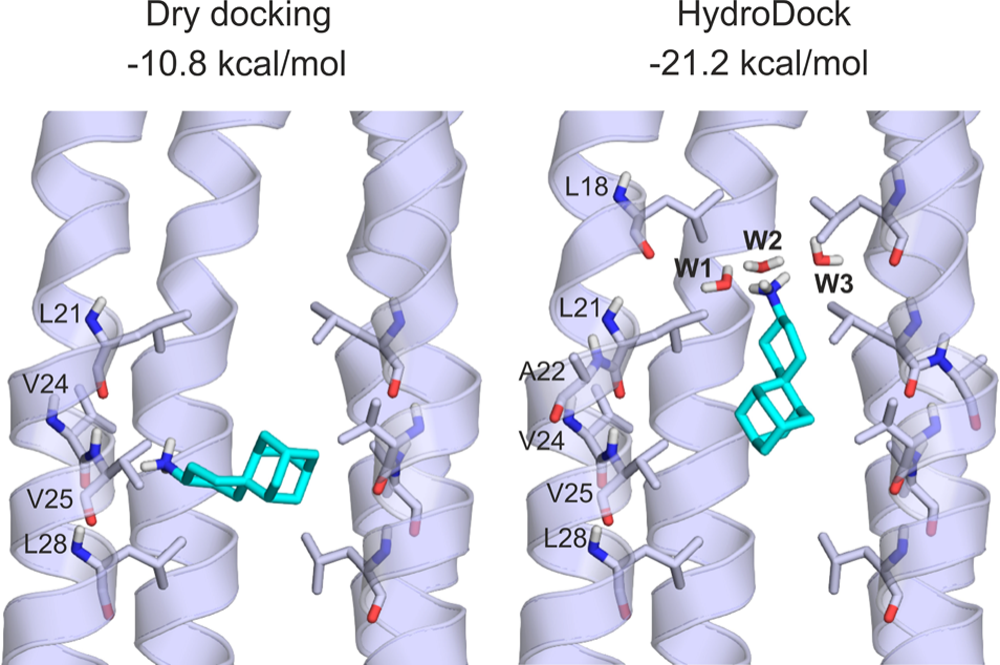
First binding mode of SA (teal sticks) to EC2 (cartoon) after dry docking (left) and HydroDock (right). Interacting amino acid residues are shown as sticks and labeled according to the 7k3g structure file. Water molecules are shown as red and white sticks and labeled as W1, ..., W3. Lennard-Jones interaction energies calculated (Methods) between the ligand SA and the (hydrated) target EC2 are shown at the top of the figure.

The abovementioned hydrophobic belts of EC2 are necessary to accommodate the hydrocarbon heads of the amphipathic AA derivatives; interfacial water molecules help in the orientation of the ligands in the EC2 channel similar to their binding modes in M2A as discussed in the previous section. For example, in the first binding mode of SA (Figure 6 and Tables S5 and S6), its spiroadamantyl group is captured in a sandwich of hydrophobic side chains arranged in several belts in the EC2 channel (Figure 6). However, the hydrophobic interactions alone are not enough to obtain the final orientation of the ligand. Dry docking positioned SA perpendicular to the helical axes of the EC2 channel, and the only H-bonding interaction was formed with a backbone amide group of V24. HydroDock refinements that introduced explicit water molecules yielded a parallel orientation, and the protonated amino group formed three H-bonds with water molecules W1, ..., W3 bridging SA with the inner wall of EC2. This bridging system of waters found by HydroDock resulted in an almost doubled SA-EC2 interaction energy if compared with dry docking (Figure 6). Similar observations can be made for the role of water molecules in the binding of ligands AA (Figure S5) and RA as well.

## Conclusions

Determination of water molecules mediating drug–target interactions is often missing or they are erroneously positioned due to inherent limitations of structure determination methods.^25^ However, the COVID-19 pandemic showed that drug repositioning or design projects often fail due to such structural errors, resulting in misprediction of drug–target interactions. The present study showed that precise positioning of interfacial water molecules is essential for correct calculation of interaction of viral channels with amphipathic ligands of the AA type. A new protocol, HydroDock, was introduced to build the hydrated target–ligand complex structures and help in the repositioning of the ligands between viral channels. In our examples, HydroDock built the hydrated complex structures from scratch and required only the apo target and ligand structures as inputs. The structures showed excellent agreements with experimental results. The atomic resolution complex structures showed that water plays a similar role in the binding of amphipathic AA derivatives to transmembrane ion channels of both influenza A (M2A) and SARS-CoV-2 (EC2). While the hydrophobic regions of the channels capture the bulky hydrocarbon group of the ligand, the surrounding waters direct its orientation parallel with the axes of the channels via bridging interactions with the ionic ligand head. Such elucidation of the role of waters is often requested,^21,25,70^ and therefore, future applications of HydroDock can be expected in the design and repositioning of drug candidates.

## Supporting Information Available

Wet docking result, wet docking of AA to M2A, the match between crystallographic reference water positions (red spheres) and the predicted water positions, the movement of RA (teal sticks) during MD simulations, the orientation of ligand AA after dry docking and HydroDock, RA1 and RA2 dry docked binding modes, comparison of holo (6BKK) and apo (3LBW) water structures, the structural fit of M2A targets, the movement of the water molecule, the statistics of generation of the ligand conformation pool, dry docked and HydroDock reprentatives of AA, RA, and SA (PDF)
Coordinate files of all snapshots of ligand binding modes, raw data of ER and IR binding regions, detailed instruction for evaluations (ZIP)

## Notes

AutoDock (http://autodock.scripps.edu/), MobyWat (http://mobywat.com/), and GROMACS (https://www.gromacs.org/) are open source programs. Schrödinger’s Maestro (https://www.schrodinger.com/products/maestro), MOPAC (http://openmopac.net/), GAMESS (https://www.msg.chem.iastate.edu/gamess/), acpype (https://pypi.org/project/acpype/), and antechamber (http://ambermd.org/antechamber/ac.html) are freely accessible programs for academic use. MOLEonline (https://mole.upol.cz/) and RED-vIII (https://upjv.q4md-forcefieldtools.org/REDServer-Development/) are accessible web servers. In-house scripts were used for average ligand structure calculation and RMSD comparisons between the average and the ligand conformations from the ligand pool and between the experimental and the ligand conformations from the ligand pool. A detailed instruction for these evaluations with a README file and the scripts are available in the Supporting Information. There is also an example for one system with all the ligand conformation pool files (sep*.pdb), the in-house scripts mentioned above, log files containing the results of the scripts, excel files created from the log files to help in evaluation, a reference pdb structure for RMSD calculation, the average ligand conformation file, and a fit.pdb file that was used for structure alignment with the gmx trjconv command line. There are three additional examples for energy minimization of the ligands in a separate folder, and all the pdb files were used to create the figures of the main text. A tutorial document, input, and output files are provided in a GitHub repository https://github.com/csabahetenyi/HydroDock.

## ABBREVIATIONS

EC2: SARS-COV-2 envelope protein E
M2A: influenza 2A transmembrane protein
