# Supplementary material for: Determination of Ligand Binding Modes in Hydrated Viral Ion Channels to Foster Drug Design and Repositioning
Source: J Chem Inf Model. 2021 Jul 27;61(8):4011–22. doi: 10.1021/acs.jcim.1c00488 (PMC8389532; doi:10.1021/acs.jcim.1c00488)
Supplement: Supplementary file 1 — ci1c00488_si_001.pdf [file ci1c00488_si_001.pdf]

Supporting Information

Determination of ligand binding modes in hydrated viral ion channels to foster drug design and repositioning

Balázs Zoltán Zsidó[1], Rita Börzsei[1,2], Viktor Szél[1], Csaba Hetényi[1,*]

[1]Pharmacoinformatics Unit, Department of Pharmacology and Pharmacotherapy, Medical School, University of Pécs, Szigeti út 12, 7624 Pécs, Hungary.
[2]Department of Pharmacology, Faculty of Pharmacy, University of Pécs, Szigeti út 12, 7624 Pécs, Hungary.

*Corresponding author, e-mail: hetenyi.csaba@pte.hu

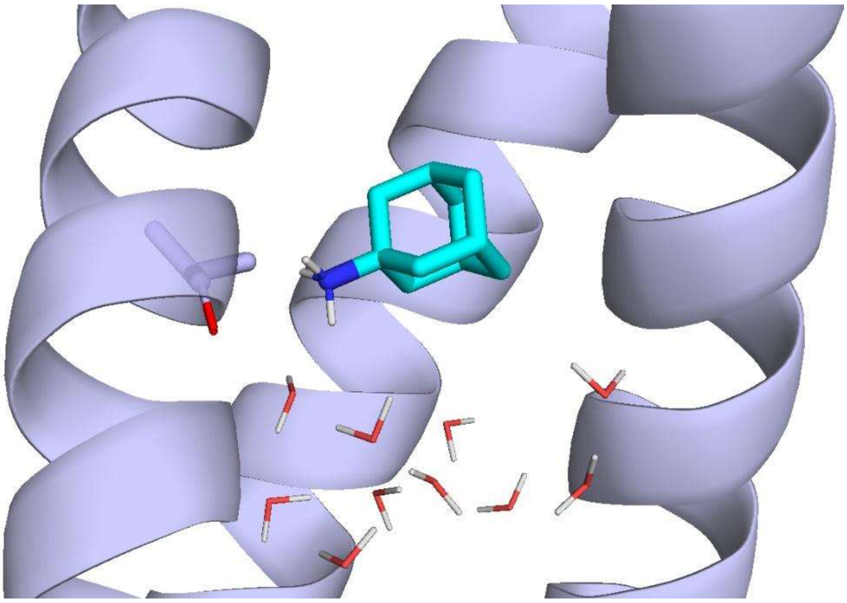

Figure S1
The wet docking result of AA to M2A after Refinement S. To include water molecules in docking experiments, H atoms were added and a two-step minimization was applied (Refinement S). After refinement, the H atoms pointed towards the experimental position of ligand AA repelling the positive charge of the aminium moiety of the ligand, resulting in a mis-docked AA. Thus, in order to refine the orientation of the H atoms, the two-step minimization in Refinement S was not enough, involvement of a short MD simulation (100 ps) was also necessary (Refinement R). M2A is shown as grey cartoon, A30 is shown as sticks, water molecules are shown as red and white thick lines and AA is shown as teal sticks.

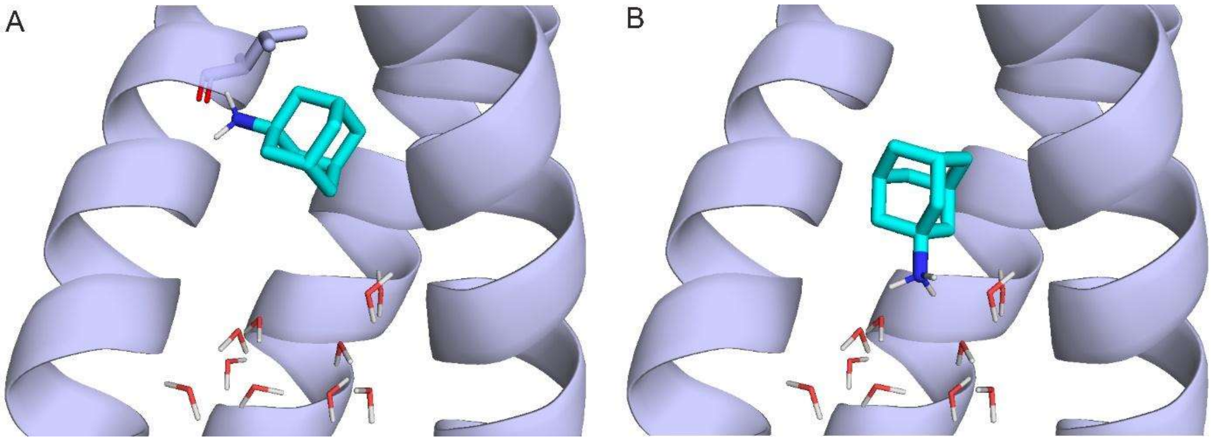

Figure S2

Wet docking of AA to M2A with Gasteiger (A) and TIP3P (B) partial charges on the water molecules. A) Although the H-bonding network of the water molecules was established properly after Refinement R, ligand AA did not get closer to its experimental binding position during docking experiments. AA coordinated to the carbonyl oxygen of V27 with its aminium moiety, instead of the water oxygens (RMSD= 3.12 Å). In this case, water molecules were equipped with Gasteiger-Marsili partial charges which was found an improper partial charge model for water molecules **B**) Using TIP3P partial charges on water molecules, ligand AA found its binding conformation close to the experimental position (**Table 3**). TIP3P water partial charges are two times greater in absolute value, than Gasteiger-Marsili ones (Table below), resulting in a larger attractive Coulomb interaction energy with the waters in the TIP3P case, which explains the differences between the docking results. Although using a uniform charge system is a standard protocol in docking calculations, in the case of water molecules, the use of TIP3P charges can be recommended even if the solutes have a different charge system like Gasteiger-Marsili. M2A is shown as grey cartoon, refined water molecules are shown as red and white thick lines, V27 is shown as sticks in A figure and AA is shown as teal sticks.

Partial charges in water	Gasteiger-Marsili	TIP3P
q(O)	-0.411	-0.820
q(H)	+0.205	+0.410

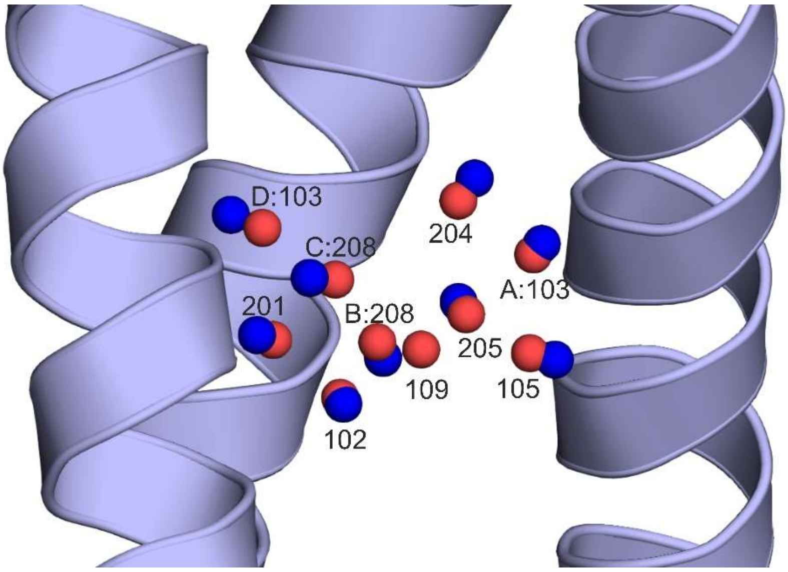

Figure S3

The match between crystallographic reference water positions (red spheres) and the predicted water positions (blue spheres) of system 6bkk in the M2A (grey cartoon) ion channel (values of deviations are listed in Table 2). An overall excellent match was found, with an exception of D:w109. This mismatch is not surprising, as the O-O distance of D:w109 and B:w208 is 2.4 Å and that of D:w109 and D:w105 is 2.6 Å indicating (non-reproducible) close contacts in the experimental structure. Water numbering follows that of PDB 6BKK.

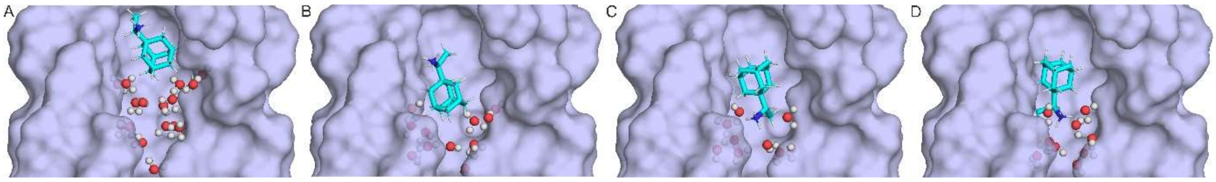

Figure S4

The movement of RA (teal sticks) during MD simulation of the 4th step of HydroDock in the M2A channel (apo). 4 frames are shown of the MD simulation: A) the starting (1st step of HydroDock, dry docked ligand binding mode), B) the 1st, C) the 100th and D) the 354th, representative binding mode after HydroDock (**Table 4**). The rotational movement of RA is seen from an initially head-to-tail binding mode to the final representative binding mode of HydroDock. During the rotation the H-bonds of the aminium moiety of RA rearrange, which is mediated by water molecules from the 2nd step of HydroDock.

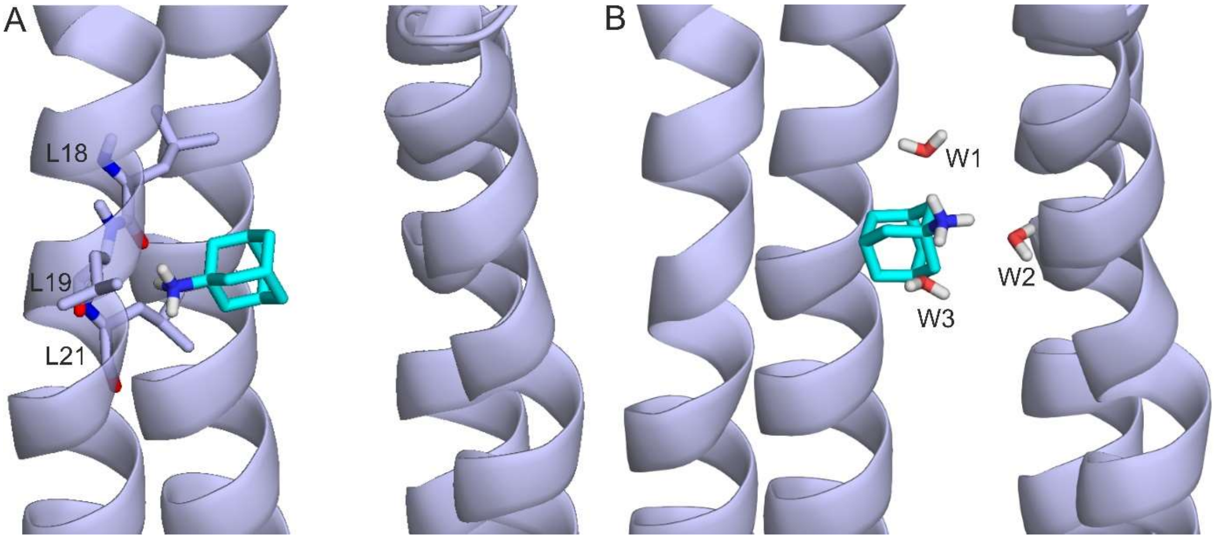

Figure S5
The orientation of ligand AA (binding mode AA3) after dry docking (A, Supp. Table 5) and HydroDock (B, Supp. Table 6). A) The representative AA3 binding mode of dry docking (step 1 of HydroDock) is H-bonded to the backbone O of L18. **B**) The representative AA3 binding mode of HydroDock forms multiple H-bonds with water molecules W1…3. The H-bonds created by the aminium moiety of AA are re-arranged during HydroDock, and energetically more favorable than those after dry docking (**Fig. 6b**). EC2 is shown as grey cartoon, interacting amino acids are shown as all atom representation grey sticks, and AA is shown as teal sticks. Water molecules are shown as red and white sticks.

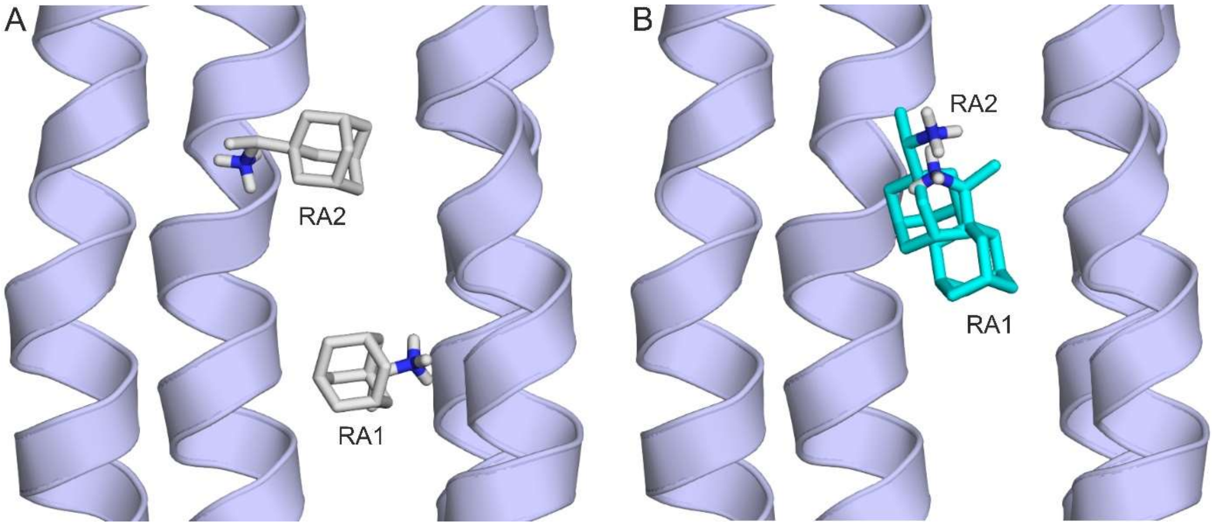

Figure S6

RA1 and RA2 dry docked binding modes converged to a common position in the EC2 channel (grey cartoon) during MD simulations in Step 4 of HydroDock. **A**) The two dry-docked binding modes of RA (grey sticks) provided two distinct starting points for the MD simulations. **B**) The two representative binding modes of RA converged during MD simulations, and from two distinct binding modes took up final binding modes that are close to each other (teal sticks). Waters are not shown.

Table S1

Comparison of holo (6BKK) and apo (3LBW) water structures. Protein superposition RMSD: 0.4 Å. Water molecules were considered identical within a match threshold of 1.0 Å, exceptions are marked. In the holo system there were 10 water oxygen positions within 5.0 Å of the ligand, when compared to the 6 water oxygens of the apo systems, which was the reason to use the holo system as reference.

Holo water #	Apo water #	Distance (Å)
A:w102	A:w100	0.5
Aw103	-	
D:w103	-	
D:w105	D:w119	0.6
D:w109	D:w122	1.9[a]
B:w201	B:w104	0.4
B:w204	-	
C:w205	C:w117	0.3
B:w208	B:w112	1.6[a]
C:w208	-	

[a]The line connecting water molecules D:w122 and B:w112 in the apo structure is turned by 90° around the vertical axis of M2A, when compared to that of the water molecules of the holo structure, which results in a slightly elevated distance.

Table S2
The structural fit of M2A targets after pair-wise alignments of protein backbone Cα atoms

	6BKK	6BKL	6BMZ
RMSD compared to 3LBW (Å)	0.39	0.39	0.22

Table S3
The movement of water molecule C:w208 during Refinements S and R

	C:w208 distance from crystallographic position (Å)	Iteration steps
Refinement S cg1*	0.9	8047
Refinement S cg2*	0.6	2013
Refinement R cg1*	0.3	1915
Refinement R md	0.2	50000
Refinement R cg2*	0.2	1794

*In cg1 and cg2, the sum values of sd1+cg1 and sd2+cg2 steps are shown, respectively (Step 2 of HydroDock in **Methods**).

Table S4

The statistics of generation of the ligand conformation pool of Table 4 (step 5 of HydroDock). The conformational pools resulted after the MD simulations (step 4 of HydroDock) contain different number of ligands each. The length of the MD simulations varies from 40 to 100 ns, ligand positions are written every 0.1 ns. Using HydroDock method we suggest a minimum of 40 ns of MD simulations (for a system of the same size as 6BKK), as after reaching this limit, the length of the MD simulation has no significant improving effect on the results in the case of drug-like ligands. For RA somewhat longer simulations were carried out compared to AA and SA, to see if the head-to-tail conformation would eventually turn over in the case of the holo system.

Ligand	M2A conformation	N_{pool}	Time (ns)	Mean (Å)[a]	SD[a]
AA	Holo	433	43.3	1.7	0.7
AA	Apo	460	46.0	1.7	0.7
RA	Holo	999	99.9	4.0	0.1
RA	Apo	826	82.6	1.7	0.7
SA	Holo	470	47.0	1.9	0.7
SA	Apo	760	76.0	1.2	0.4

[a]Mean and SD of the RMSD values of each member of the conformational pool compared to their respective experimental ligand position are shown. Note that in Table 4, the comparison is done to the respective average ligand positions.

Table S5
Dry docked reprentatives of AA, RA and SA with list of EC2 residues of closest heavy atom distances ≤5.0 Å measured from the ligands (step 1 of HydroDock).

Binding mode	Docking box code*	N15 flexibility	EC2 amino acid (closest distance, Å)
AA1	Box A	OFF	T11 (3.5), L12 (34.1), V14 (3.5), N15 (2.8)
AA2	Box A	OFF	T11 (4.5), L12 (4.2), V14 (3.9), N15 (2.8)
AA3	Box A	OFF	N15 (4.9), L18 (2.8), L19 (3.4), F20 (4.7), L21 (3.3), A22 (4.5)
AA4	Box B	ON	V14 (4.7), N15 (2.8), S16 (4.8), L18 (3.4)
AA5	Box B	ON	V14 (4.8), N15 (4.6), L18 (4.0), L21 (4.5)
RA1	Box B	OFF	L21 (4.2), A22 (5.0), V24 (3.9), V25 (3.3), L28 (3.2)
RA2	Box B	OFF	L18 (3.6), L19 (4.0), F20 (5.0), L21 (3.0), A22 (4.7)
SA1	Box B	OFF	L21 (5.0), V24 (3.0), V25 (3.2), L28 (3.0)

*Box A: The box (edge size: 0.375 × 90) covered the extra-viral end of EC2 down to the middle of EC2 10.991 -0.074 -0.009 in PDB structure 7k3g. Box B: The box covered the entire surface of EC2.

Table S6
HydroDock reprentatives of AA, RA and SA with list of EC2 residues of closest heavy atom distances ≤5.0 Å measured from the ligands

Binding mode	EC2 amino acid (closest distance, Å)
AA1 [a]	T11 (4.8), L12 (4.2), I13 (4.4), V14 (3.9), N15 (3.8)
AA2 [b]	T11 (4.5), L12 (3.8), N15 (4.9)
AA3	L18 (4.3), L19 (4.4), L21 (3.8)
AA4	L12 (4.6), V14 (4.9), N15 (3.0), L18 (4.3)
AA5	L18 (3.7), L19 (4.5), L21 (4.4)
RA1	L18 (3.7), L21 (4.4), A 22 (4.9), V24 (4.6), V25 (3.9)
RA2	L18 (4.9), L21 (4.0), V25 (4.7)
SA1	L18 (4.4), L21 (4.3), A22 (5.0), V24 (4.9), V25 (4.1), L28 (5.0)

[a] Dissociated after 23.6 ns, the representative structure was selected from the first 236 MD snapshots.
[b] Dissociated after 31.8 ns, the representative structure was selected from the first 318 MD snapshots.
